# Factors associated with the intention of Syrian adult population to accept COVID19 vaccination: a cross-sectional study

**DOI:** 10.1186/s12889-021-11361-z

**Published:** 2021-07-04

**Authors:** Okbah Mohamad, Ali Zamlout, Naseem AlKhoury, Abd Aljawad Mazloum, Marah Alsalkini, Rafea Shaaban

**Affiliations:** 1 Faculty of Medicine, Tishreen University, Latakia, Syria; 2 Faculty of Medicine, Albaath University, Homs, Syria; 3 Faculty of Medicine, Al-Andalus University for Medical Sciences, Tartus, Syria

**Keywords:** COVID-19, Vaccine hesitancy, Syria, Pandemic

## Abstract

**Background and objectives:**

With global efforts to develop and deliver a COVID-19 vaccine rapidly, vaccine hesitancy stands as a barrier to these efforts. We aimed to estimate the proportion of Syrian adult population intending to be vaccinated against COVID-19 and, principally, to assess the demographic and attitudinal factors associated with it in order to approach suitable solutions.

**Methods:**

An anonymous online questionnaire was conducted between 23rd December 2020 and 5th January 2021 in various provinces in Syria. A total of 3402 adults were sampled to reflect the population demographic factors. Attitudinal factors included Covid-19 fears, risks, and beliefs on the origin. Vaccination hesitancy and knowledge were also measured. The intention to get vaccinated against COVID-19 was the primary endpoint.

**Results:**

According to their statements, 1222 participants (35.92%) will consent to get vaccinated against COVID-19. Our findings indicate that male gender, younger age, rural residence, not having children, smoking, fear about COVID-19, individual perceived severity, believing in the natural origin of the coronavirus, and high vaccination knowledge were positive predictors of embracing COVID-19 vaccine when it is available.

**Conclusion:**

COVID-19 vaccine acceptance rate is considerably poor across Syrian population compared to populations in developed countries. Vaccine hesitancy is closely bound to the fear of side effects and doubts about vaccine efficacy. Factors such as conspiracy beliefs and myths about the vaccine lower vaccine uptake. Thus, interventional educational campaigns are increasingly required to overcome misinformation and avert low vaccination acceptance rates.

## Introduction

The outbreaks in human history are old and tragic. Diseases such as polio, smallpox, and rabies were once significant threats to our existence, but the discovery of vaccines was a breakthrough by reducing the incidence rates of such diseases [1]. According to the World Health Organization (WHO), vaccination averts 2–3 million deaths a year [2]. Moreover, since 1990, there has been a 61% decline in mortality rate among children between five and nine years old due to a decline in infectious diseases [3].

Till 14th February 2021 around 107 million cases of coronavirus disease 2019 (COVID-19) and 2 million deaths have been reported worldwide [4]. This newly emerging pandemic overwhelmed medical facilities and overloaded the burden on healthcare systems. The situation is more strenuous in war-torn Syria, where the preexisting burden on the declining healthcare system surged due to the outbreak of COVID-19. The pandemic impact was prominent in terms of overloaded hospitals, insufficient resources, and poor surveillance systems [5, 6].

To lessen the COVID-19 burden on population health concerning both death rates and exhausting healthcare systems, an effective vaccine must be developed and distributed soon. Recently, multiple vaccines have been industrialized and approved for emergency use in a relatively short time compared to other vaccines developed in the past. Namely, Oxford-AstraZeneca, Pfizer-BioNTech, and Moderna COVID-19 vaccines [7, 8]. Besides vaccine availability, effectiveness, and safety, it is important that the vaccine is tolerable by the targeted population [9].

Vaccine hesitancy raises challenges to public health agencies in order to achieve a sufficient degree of immunization necessary to protect vulnerable individuals [9]. The WHO defines vaccine hesitancy as the rejection or delay in vaccine uptake in the presence of an existing vaccine [2]. An example of such a challenge in public health is the report published by the Centre for Disease Control and Prevention in the United States that assessed the prevalence of seasonal influenza vaccine hesitancy in up to 50% of the study population [10].

This study aims to (i) estimate the intention to get vaccinated against COVID-19 among the adult Syrian population, (ii) determine demographic and attitudinal factors associated with participants intentions, and (iii) measure the population’s general knowledge about vaccinations.

## Methods

### Sample and procedure

A cross-sectional study collected data through an anonymous online questionnaire from Social Media networks, including Facebook, Twitter, and Telegram, covering the period between 23rd December 2020 and 5th January 2021 among the adult general population, while covering various provinces in Syria. We developed a standardized questionnaire based on a literature review [11–15].

### Questionnaire

All questions were administered in Arabic language as Arabic is the native language of the Syrian population.

### Demographic questionnaire

The demographic questionnaire collected data on age, gender, geographic location, region of residence (urban or rural), educational degree, employment status before and during COVID-19 pandemic, working in the healthcare sector, marital status, parenthood status, smoking status, and if the participant had chronic medical conditions.

### Coronavirus questionnaire

This section addressed: (1) Fears about COVID-19, (2) Perceived risk of contracting COVID-19, (3) Perceived severity of COVID-19 on your life, (4) Previous COVID-19 infection, (5) Beliefs on coronavirus origin, and (6) Intention to get vaccinated when COVID-19 vaccine is available in Syria on a 5-level Likert scale: 1 (strongly disagree), 2 (disagree), 3 (undecided), 4 (agree), or 5 (strongly agree). Responders declining the COVID-19 vaccine option or undecided yet were automatically directed to a specific question addressing reasons for refusing the vaccine. Participants could choose one or more of the following statements: “Fear of side effects”, “Doubts about the efficiency of the vaccine”, “The vaccine is not important”, “This is not the right time”, “I just refuse the vaccine”, and “other reasons”.

### Vaccine hesitancy

Vaccine hesitancy was evaluated using three questions: “Have you ever refused a vaccine for yourself or a child because you considered it as useless or dangerous?” “Have you ever postponed a vaccine recommended by a physician because of doubts about it?” “Have you ever had a vaccine for a child or yourself despite doubts about its efficacy” [16]. If a participant answered yes to any of these proposals, he or she was considered to be “vaccine hesitant”.

### Knowledge about vaccines

This section evaluated participants’ knowledge about vaccines with nine statements, each one providing 3 options: correct, incorrect, or do not know [17]. “Incorrect” or “do not know” options were scored as zero, and “correct” was scored as one. Therefore, higher scores indicated better knowledge of vaccines.

The last section of the questionnaire presented people with questions on how often they used the following sources for gaining information about vaccinations: healthcare providers (doctor, nurse, ...), the internet and media, and reliable sources (WHO website, medical journals, …). Possible answers ranged from never (1) to always (6).

### Statistical analysis

Data were entered in Microsoft Excel software and the statistical analysis was carried out using SPSS version 25.0. Descriptive statistics of the raw data were presented as frequencies and percentages. We combined responses regarding COVID-19 vaccine acceptance into two categories: 1 (Agree), or 0 (Disagree), and ran multiple binomial logistic regression (multivariate analysis) to model demographic and attitudinal factors predictive of participants’ willingness to get vaccinated against COVID-19. We considered *P* value < 0.05 to be significant.

## Results

There were a total of 3402 participants who completed the online questionnaire (Table 1). The response rate cannot be calculated as participants are recruited based on open invitations via social networks that do not provide information on people who viewed the online post.

**Table 1:** Association between participants characteristics and their acceptance of COVID19 vaccine

Variable	Category	Total N(%)	Do Not Agree N(%)	Agree N(%)	Chi-Square
Sex	Male	1219 (35.83)	656 (53.81)	563 (46.19)	< 0.0001
	Female	2183 (64.17)	1524 (69.81)	659 (30.19)	
Age	> 30	1732 (50.91)	1196 (69.05)	536 (30.95)	< 0.0001
	18–30	1670 (49.09)	984 (58.92)	686 (41.08)	
Residency province	West	1376 (40.45)	880 (63.95)	496 (36.05)	0.299
	Northern-East	265 (7.79)	162 (61.13)	103 (38.87)	
	Middle	594 (17.46)	369 (62.12)	225 (37.88)	
	South	1167 (34.3)	769 (65.9)	398 (34.1)	
Region of residence	Rural	973 (28.6)	596 (61.25)	377 (38.75)	0.03
	Urban	2429 (71.4)	1584 (65.21)	845 (34.79)	
Marital status	Married	1690 (49.68)	1168 (69.11)	522 (30.89)	< 0.0001
	Not married	1712 (50.32)	1012 (59.11)	700 (40.89)	
Having kids	Yes	1535 (45.1)	1068 (69.58)	467 (30.42)	< 0.0001
	None	1867 (54.9)	1112 (59.56)	755 (40.44)	
Highest level of education	Post-graduate	545 (16.02)	346 (63.49)	199 (36.51)	0.184
	School	492 (14.46)	334 (67.89)	158 (32.11)	
	High Education	2272 (66.78)	1436 (63.2)	836 (36.8)	
	None	93 (2.73)	64 (68.82)	29 (31.18)	
Pre-coronavirus employment status	Employed	1996 (58.67)	1278 (64.03)	718 (35.97)	0.94
	Not employed	1406 (41.33)	902 (64.15)	504 (35.85)	
Employment change due to coronavirus	Newly employed	114 (3.35)	71 (62.28)	43 (37.72)	0.453
	Furlough	148 (4.35)	86 (58.11)	62 (41.89)	
	Newly Unemployed	187 (5.5)	121 (64.71)	66 (35.29)	
	None	2953 (86.8)	1902 (64.41)	1051 (35.59)	
Financial income	Salary + Other	682 (20.05)	439 (64.37)	243 (35.63)	0.108
	Freelancing	398 (11.7)	241 (60.55)	157 (39.45)	
	Family	1354 (39.8)	853 (63)	501 (37)	
	Salary only	968 (28.45)	647 (66.84)	321 (33.16)	
Healthcare worker	Student	526 (15.46)	282 (53.61)	244 (46.39)	< 0.0001
	Yes	449 (13.2)	268 (59.69)	181 (40.31)	
	No	2427 (71.34)	1630 (67.16)	797 (32.84)	
Smoking status	Yes	1509 (44.36)	930 (61.63)	579 (38.37)	0.008
	No	1893 (55.64)	1250 (66.03)	643 (33.97)	
Chronic medical conditions	Yes	464 (13.64)	283 (60.99)	181 (39.01)	0.136
	No	2938 (86.36)	1897 (64.57)	1041 (35.43)	
Fears about COVID-19	Yes	2229 (65.52)	1341 (60.16)	888 (39.84)	< 0.0001
	No	1173 (34.48)	839 (71.53)	334 (28.47)	
Perceived risk of contracting COVID-19	Likely	3243 (95.33)	2068 (63.77)	1175 (36.23)	0.087
	Unlikely	159 (4.67)	112 (70.44)	47 (29.56)	
Perceived severity of effect of COVID-19 to one’s life	Serious	1049 (30.83)	626 (59.68)	423 (40.32)	< 0.0001
	Not serious	2353 (69.17)	1554 (66.04)	799 (33.96)	
Had COVID-19?	Yes, a positive test	170 (5)	112 (65.88)	58 (34.12)	0.079
	No, a negative test	1646 (48.38)	1066 (64.76)	580 (35.24)	
	May have had it but not been tested	161 (4.73)	88 (54.66)	73 (45.34)	
	Not had it but not been tested	1425 (41.89)	914 (64.14)	511 (35.86)	
Beliefs on the origin of the virus	Artificial/ Do not know	2711 (79.69)	1902 (70.16)	809 (29.84)	< 0.0001
	Natural	691 (20.31)	278 (40.23)	413 (59.77)	
General Vaccine Hesitancy	Not hesitant	2545 (74.81)	1622 (63.7)	923 (36.3)	0.467
	Hesitant	857 (25.19)	558 (65.1)	299 (34.9)	
Vaccination Knowledge	0	126 (3.7)	107 (84.92)	19 (15.08)	< 0.0001
	1	168 (4.94)	136 (80.95)	32 (19.05)	
	2	371 (10.91)	290 (78.17)	81 (21.83)	
	3	507 (14.9)	364 (71.79)	143 (28.21)	
	4	489 (14.37)	330 (67.48)	159 (32.52)	
	5	429 (12.61)	272 (63.4)	157 (36.6)	
	6	436 (12.82)	252 (57.8)	184 (42.2)	
	7	361 (10.61)	187 (51.8)	174 (48.2)	
	8	280 (8.23)	143 (51.07)	137 (48.93)	
	9	235 (6.91)	99 (42.13)	136 (57.87)	
Healthcare provider	Never	467 (13.73)	319 (68.31)	148 (31.69)	< 0.0001
	Occasionally	197 (5.79)	145 (73.6)	52 (26.4)	
	Sometimes	276 (8.11)	187 (67.75)	89 (32.25)	
	Often	437 (12.85)	283 (64.76)	154 (35.24)	
	Usually	554 (16.28)	363 (65.52)	191 (34.48)	
	Always	1471 (43.24)	883 (60.03)	588 (39.97)	
Media	Never	991 (29.13)	652 (65.79)	339 (34.21)	0.594
	Occasionally	612 (17.99)	396 (64.71)	216 (35.29)	
	Sometimes	490 (14.4)	317 (64.69)	173 (35.31)	
	Often	555 (16.31)	343 (61.8)	212 (38.2)	
	Usually	341 (10.02)	210 (61.58)	131 (38.42)	
	Always	413 (12.14)	262 (63.44)	151 (36.56)	
Reliable resources	Never	774 (22.75)	548 (70.8)	226 (29.2)	< 0.0001
	Occasionally	275 (8.08)	183 (66.55)	92 (33.45)	
	Sometimes	364 (10.7)	245 (67.31)	119 (32.69)	
	Often	469 (13.79)	311 (66.31)	158 (33.69)	
	Usually	521 (15.31)	327 (62.76)	194 (37.24)	
	Always	999 (29.37)	566 (56.66)	433 (43.34)	

Most participants were female (64.2%). There were 49.1% participants aged 18–30, and 50.9% aged 31 or above. For health status, 13.6% of them reported having chronic conditions. 71.4% of respondents were residing in urban regions. Regarding work-related characteristics, 58.7% of them were working before the pandemic, while 5.5% lost their job due to coronavirus. Vaccine hesitancy was observed in 857 (25.2%) respondents. 2229 (65.5%) respondents had fears about COVID-19, 3243 (95.3%) considered themselves at risk of contracting COVID-19, and 1049 (30.8%) perceived COVID-19 as being serious to their own lives (Table 1).

According to their statements, 1222 participants (35.92%) will consent to get vaccinated against COVID-19. In multivariable analysis (Table .2), male gender, younger age, rural residence, not having children, smoking, fear about COVID-19, individual perceived severity, believing in the natural origin of the coronavirus, and high vaccination Knowledge remained associated with COVID-19 vaccine acceptance. Surprisingly, no significant influence arose from studying or working within the medical field on respondents’ acceptance or rejection of COVID-19 vaccine.

**Table 2:** Factors associated with intentions to accept COVID-19 vaccination when it is available

	Crude OR (95%CI)	P value	Adjusted OR (95%CI)	P value
Sex				
Female	0.50 (0.44, 0.58)	< 0.0001	0.53 (0.44, 0.62)	< 0.0001
Male	Reference		Reference	
Age				
18–30	1.56 (1.35, 1.79)	< 0.0001	1.32 (1.06, 1.64)	0.012
>/30	Reference		Reference	
Residency province				
Northern-East	1.13 (0.86, 1.48)	0.38	1.09 (0.8, 1.49)	0.57
Middle	1.08 (0.89, 1.32)	0.44	1.07 (0.86, 1.33)	0.57
South	0.92 (0.78, 1.08)	0.31	0.93 (0.78, 1.12)	0.45
West	Reference		Reference	
Region of residence				
Urban	0.84 (0.72, 0.98)	0.03	0.82 (0.69, 0.97)	0.018
Rural	Reference		Reference	
Marital status				
Not married	1.55 (1.34, 1.78)	< 0.0001	0.95 (0.68, 1.34)	0.79
Married	Reference		Reference	
Having kids				
No	1.55 (1.35, 1.79)	< 0.0001	1.40 (1.00, 1.96)	0.05
Yes	Reference		Reference	
Highest level of education				
School	0.82 (0.64, 1.06)	0.137	1 (0.74, 1.36)	0.99
High Education	1.01 (0.83, 1.23)	0.9	1.03 (0.83, 1.3)	0.77
None	0.79 (0.49, 1.26)	0.32	0.92 (0.52, 1.62)	0.77
Post-graduate qualification	Reference		Reference	
Pre-coronavirus employment status				
Not employed	0.995 (0.86, 1.15)	0.94	0.87 (0.66, 1.14)	0.32
Employed	Reference		Reference	
Employment change due to coronavirus				
Furlough	1.19 (0.72, 1.96)	0.495	1.26 (0.71, 2.22)	0.43
Newly Unemployed	0.9 (0.56, 1.46)	0.67	0.77 (0.44, 1.35)	0.36
None	0.91 (0.62, 1.34)	0.64	0.86 (0.56, 1.34)	0.51
Newly employed	Reference		Reference	
Financial income				
Freelancing	1.18 (0.91, 1.52)	0.21	1.24 (0.92, 1.66)	0.16
Family	1.06 (0.88, 1.29)	0.54	1.14 (0.84, 1.55)	0.4
Salary only	0.9 (0.73, 1.1)	0.298	0.99 (0.79, 1.24)	0.92
Salary + Other	Reference		Reference	
Healthcare worker				
Yes	0.78 (0.61, 1.007)	0.057	1.07 (0.77, 1.48)	0.70
No	0.57 (0.47, 0.68)	< 0.0001	1.27 (0.97, 1.66)	0.082
Student in healthcare	Reference		Reference	
Smoking status				
No	0.83 (0.72, 0.95)	0.008	0.79 (0.68, 0.93)	0.005
Yes	Reference		Reference	
Chronic medical conditions				
No	0.86 (0.7, 1.05)	0.136	0.88 (0.7, 1.11)	0.28
Yes	Reference		Reference	
fears about COVID-19				
No	0.60 (0.52, 0.70)	< 0.0001	0.54 (0.46, 0.64)	< 0.0001
Yes	Reference		Reference	
Perceived risk of contracting COVID-19				
Unlikely	0.74 (0.52, 1.05)	0.088	0.83 (0.54, 1.25)	0.37
Likely	Reference		Reference	
Perceived severity of effect of COVID-19 on own life				
Not serious	0.76 (0.66, 0.88)	< 0.0001	0.78 (0.65, 0.92)	0.004
Serious	Reference		Reference	
Beliefs on the origin of the virus				
Natural	3.49 (2.94, 4.15)	< 0.0001	2.56 (2.11, 3.1)	< 0.0001
Artificial	Reference		Reference	
General Vaccine Hesitancy				
Hesitant	0.94 (0.8, 1.11)	0.467	1.01 (0.85, 1.21)	0.88
Not hesitant	Reference		Reference	
Knowledge	1.24 (1.20, 1.28)	< 0.0001	1.23 (1.19, 1.28)	< 0.0001

**Fig. 1:**
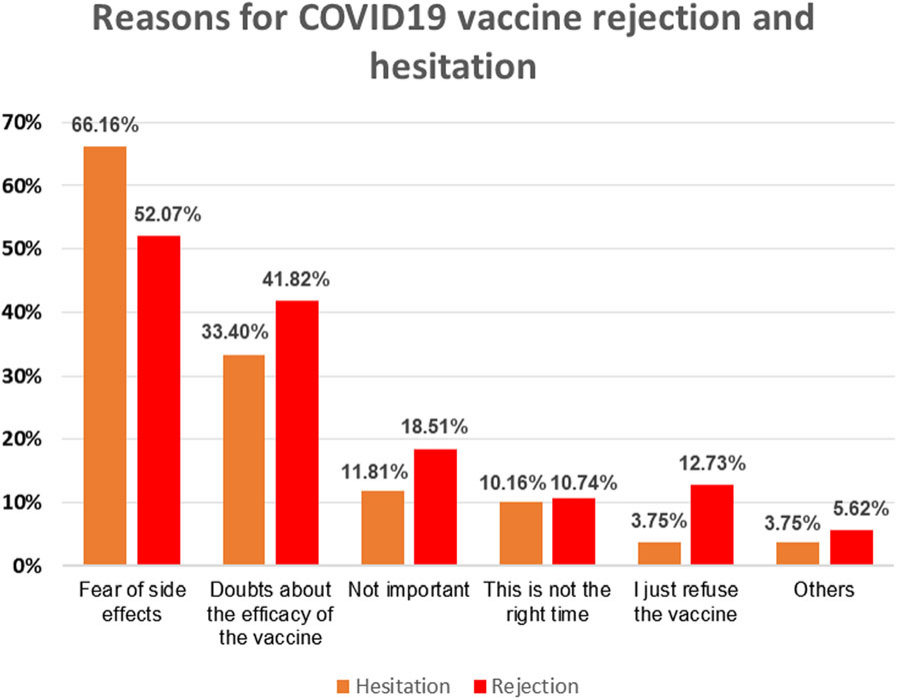
Reasons for COVID-19 vaccine rejection and hesitation

The most common reason for refusal and hesitancy to accept COVID-19 vaccination was “fear of side effects”, and other reasons included “doubts about vaccine efficiency”, “the vaccine is not important”, “not the right time to be vaccinated”, and “just refuse the vaccine” (Fig. 1).

## Discussion

This study was conducted during the second wave of COVID-19 pandemic in Syria and after the emergence of multiple promising vaccines around the world.

**Fig. 2:**
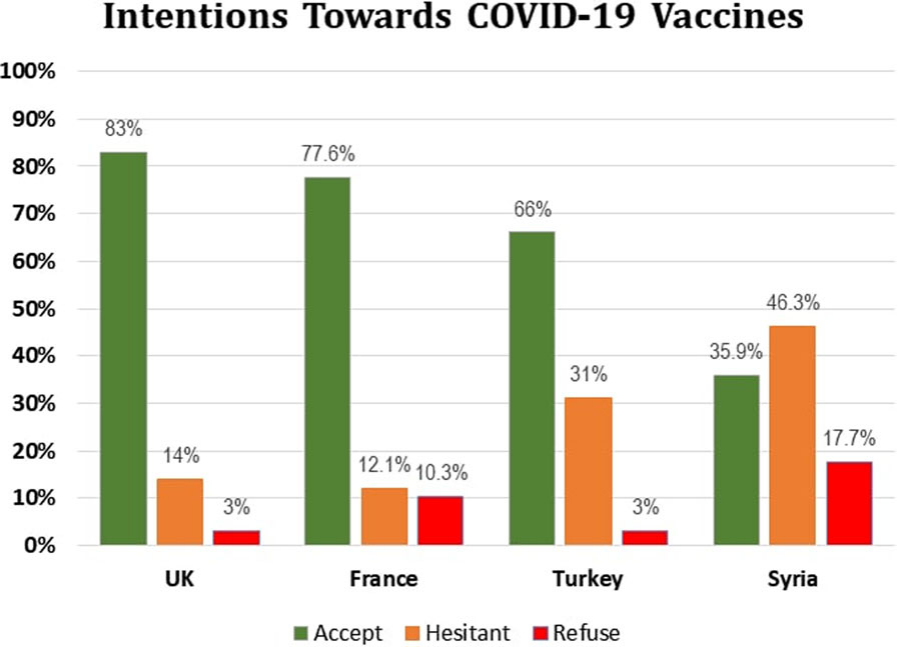
Intentions to get COVID-19 vaccination among different countries

Despite the relatively difficult experience of our population with the pandemic and its severe socioeconomic consequences, the results describe 17% refusing vaccination, 35% accepting, and 46% undecided. This level of acceptance rate is considerably low in comparison with other countries (Fig. 2) [12, 18]. In this regard, we will discuss the factors that may have played a role in these findings, the short and long-term repercussions of such numbers, and propose solutions to improve vaccine acceptance rates.

### Factors associated with vaccination intentions

According to our data, gender as a predictor of willingness for vaccination against SARS-CoV-2 presented a statistically significant difference. Males were more likely to accept the vaccine than females, which could be attributed to the higher sex-based risks of complications and mortality, in addition to the potential economic impacts of the pandemic on the father’s role as a primary source of household income [19, 20]. Whereas females were more reluctant to be vaccinated than males, as they tend to collect medical information from various sources when it comes to their families’ health [20].

The results also indicate that older people – who are also at greater risk of complications and mortality [21] – were more likely to refuse COVID-19 vaccine than younger adults. Their concerns about vaccine safety may be the reason for this disparity. On the other hand, younger people are more exposed to educational campaigns and scientific discussions related to vaccine development protocols due to their higher literacy skills [22, 23]. This leads us to think about the importance of targeting the elderly with sensitizing campaigns in order to enhance their willingness for vaccination.

Individuals who had children were more reluctant to be vaccinated compared to those who did not have children. Misconceptions alongside the fear of consequences of vaccination or leaving family members behind may explain this reluctance [24].

Interestingly, residents in rural areas were more willing to get vaccinated than urban residents. The reason for this disparity could be attributed to the online distribution method of the questionnaire that may have reached more educated groups of rural populations than disadvantaged ones, which in turn skewed the results toward acceptance. Furthermore, our data was unable to reveal a statistically significant difference between healthcare workers and the general population in terms of vaccine acceptance. This finding needs to be examined carefully, as their higher literacy skills on healthcare-related issues and recent guidelines do not seem to be a significant factor in their willingness for vaccination as expected. The former is particularly important due to the fact that the already devastated healthcare system in Syria dealing with a pandemic of this size without taking preventive measures such as vaccination could result in calamitous consequences.

In Syria, the acceptance rates were almost half compared to studies conducted in other countries (Fig. 2) [12, 18]. One of the most important pillars to whether to get vaccinated or not is the individual’s knowledge of both the disease and the role of immunization in stopping its spread. Conspiracy beliefs and misinformation were one of the main obstacles to adhere to prevention measures at the early stages of the pandemic, and the same thing is happening now with the educational programs about the importance of vaccination and herd immunity [25, 26]. Despite the scientific consensus on the natural origin of the virus [27], as well as the sensitizing campaigns and social network algorithms to encounter misinformation, only 20.31% of participants in our survey believed in the natural origin of the coronavirus.

The roots of these beliefs can be traced back to several factors. For instance, cognitive biases – like “confirmation bias”, “The availability Heuristic Bias”, “Belief biases” – make the idea of the artificial origin of the virus easier and more appealing to believe [28]. Information sources play a major role in building these beliefs among individuals [29]; participants in our survey who depend on reliable scientific sources and healthcare providers were more willing to accept the vaccine. This focuses on the importance of credible information, especially during the rapid changes and the dissemination of faulty news.

The above is particularly important because cognitive biases, the declining socioeconomic status (SES), and the collapsed healthcare system are among the most prominent features of the nearly decade-old ongoing war; this increases the randomness of the course of the pandemic in such war-torn countries compared to the rest of the world. On the other hand, making use of these factors to enhance the population’s acceptance of the vaccine is also possible, as the fear of contracting COVID-19 alongside the individual’s perception of the seriousness and consequences of infection is associated with increased willingness to accept the vaccine, as shown in our analysis.

### The special case of Syria

From a broader perspective, hesitation or rejection of the vaccine, together with the aforementioned factors, would constitute a vicious cycle in which the already devastated SES and healthcare systems would take turns reactivating it. The deteriorating SES – represented by overcrowded communities suffering from food shortages, low immunity, lack of proper education, and underprivileged healthcare settings – will make of these communities a suitable milieu for misleading information and conspiracy beliefs; thus, reducing the willingness to be vaccinated and increasing the rates of infection spread [30–32], which puts additional burdens on the already-weakened healthcare systems [31, 33]. On the other hand, the overwhelmed healthcare units will be less able to manage patients and monitor new cases, which in turn, will negatively affect the economy and population’s trust in their healthcare systems [31, 34, 35]; and thus, activating the cycle again. Moreover, logistical barriers to vaccination also play an important role; At a time when purchasing power, electricity, internet access, and fuel are scarce, logistics can prohibit vaccines from reaching individuals willing to get vaccinated. Even though approaching such a scenario in a precise statistical framework is not applicable with our study design under the rapid changes in SES as well as lack of national demographic references that can be used as statistical variables, we can conclude by observing several experiences of war-torn countries, including Syria, that the problem does not stop here, but it may rather reach the reemergence of nearly eliminated diseases [31], such as the spread of polio from Syria to Iraq between 2013 and 2014 [36, 37]. This opens the door for many questions about surveillance and management during the crisis:

In the event of an epidemic re-emergence or a mutation arising in the virus, what is the possibility of tracking new cases before spreading to other countries? If global herd immunity is reached prior to Syria, would an isolation of the country be mandated? And if so, what repercussions would Syria encounter while dealing with the successive waves of the pandemic?

Our data stop on the boundaries of these questions and cannot answer them, but we emphasize the importance of taking these points into consideration when health policymakers make relevant decisions – since one of the most important lessons learned from the world’s experience with the pandemic is that “time of action matters” [38].

### Suggested solutions

After the massive spread of the pandemic in almost all countries of the world, it became clear that the solution is only possible through achieving herd immunity [9], which requires immunizing 55–80% of the population against the virus – as a recent study showed [39]. But in order to reach this threshold, there should be a level in which the population is prepared to achieve a sufficient degree of “scientific citizenship” that in turn increases willingness for vaccination [40].

Scientific citizenship implies creating a climate of communication between the scientific community and citizens on the basis of transparency, mutual trust, and active engagement, through which it is possible to confront misinformation and enhance community confidence in their healthcare institutions [25, 26, 40]. This includes: (A) Conducting educational campaigns that discuss individuals’ concerns about the pandemic and vaccine safety, as well as explaining recommendations through sensitizing dialogues [40]. (B) Emphasizing the prosocial benefits of immunization, as several studies have demonstrated that clarifying the social benefits of herd immunity increases the willingness for vaccination [41, 42] – particularly since the experience of locking down the country during the first wave had severely affected the SES of the population [20, 43]. (C) Supporting psychological research that investigates community behavior and its perspective about vaccines. (D) Interventions should be directed to encourage health workers and elderly people with chronic diseases to get vaccinated, which contributes to relieving pressure on health care units [13].

### Strengths and limitations

To the best of our knowledge, this is the first study to investigate COVID-19 vaccine acceptance rates in Syria. The importance of this study comes from the fact it sheds light on a global problem in a war-torn developing country — and thus provides health policymakers with input in order to better steer actions, and can also be used as a simulation for other countries that have not yet investigated their population’s acceptance rates.

A key limitation of our study is the use of an online sampling method, which introduced sampling bias and hampered the generalizability despite the large sample size. Participants who do not have any educational degree formed a low proportion of our sample, possibly because they do not have access to social media networks, so future studies may use better approaches to accommodate for this category. Second, the cross-sectional design of this study precludes our ability to infer causality among dependent and independent variables. Third, this study is based on self-reported measures, which might contribute to social desirability bias and justify the high percentage of respondents who did not yet decide whether to endorse or reject the vaccine. Forth, the online sampling method implied some limitations such as the roughly low number of elderly, illiterate participants and citizens living in conflict areas like the Northeast provinces where Internet access is not constantly available. However, the proportion of these groups in the population is relatively low and the questionnaire surveyed a homogeneous sample from the various categories of the Syrian population within a short period of time, without making any modifications to the questionnaire or the methodology during the experiment. Therefore, no adjusted weights were applied to the variables.

## Conclusion

Hesitation about COVID-19 vaccine has become the most prominent problem facing health organizations at the present time of the pandemic, and it is clear that efforts must be combined to improve acceptance rates. Among the factors we studied in this paper, male gender, younger age, rural residence, not having children, smoking, fear about COVID-19, individual perceived severity, believing in the natural origin of the coronavirus, and high vaccination knowledge had a positive role in our population’s acceptance rates for vaccination. The rates may differ from one country to another or from a certain pandemic stage to another within the same country, but for developing or war-torn countries, further potential burdens exist and should be taken into consideration while discussing measures to exit the current crisis on an international scale. This study focused on Syria, but at the same time it provides an insight into countries with similar conditions, which unfortunately do not have enough epidemiological reports to coordinate international efforts to help them.

## Data Availability

The data analyzed during the current study are available from the corresponding author on reasonable request.
